# The Effect of Welding Current and Electrode Force on the Heat Input, Weld Diameter, and Physical and Mechanical Properties of SS316L/Ti_6_Al_4_V Dissimilar Resistance Spot Welding with Aluminum Interlayer

**DOI:** 10.3390/ma14051129

**Published:** 2021-02-27

**Authors:** Iqbal Taufiqurrahman, Azlan Ahmad, Mazli Mustapha, Turnad Lenggo Ginta, Luthfi Ady Farizan Haryoko, Imtiaz Ahmed Shozib

**Affiliations:** 1 Mechanical Engineering Department, Universiti Teknologi PETRONAS, Seri Iskandar 32610, Perak Darul Ridzuan, Malaysia; mazli.mustapha@utp.edu.my (M.M.); luthfi_17006010@utp.edu.my (L.A.F.H.); ahmed_19001670@utp.edu.my (I.A.S.); 2 PT. Orca Industri Akademi, Kuningan, Jakarta 12950, Indonesia; turnad@orca-institute.com

**Keywords:** resistance spot welding, stainless steel 316L, Ti_6_Al_4_V alloy, mechanical properties, aluminum interlayer, welding current, heat input

## Abstract

Welding parameters obviously determine the joint quality during the resistance spot welding process. This study aimed to investigate the effect of welding current and electrode force on the heat input and the physical and mechanical properties of a SS316L and Ti_6_Al_4_V joint with an aluminum interlayer. The weld current values used in this study were 11, 12, and 13 kA, while the electrode force values were 3, 4, and 5 kN. Welding time and holding time remained constant at 30 cycles. The study revealed that, as the welding current and electrode force increased, the generated heat input increased significantly. The highest tensile-shear load was recorded at 8.71 kN using 11 kA of weld current and 3 kN of electrode force. The physical properties examined the formation of a brittle fracture and several weld defects on the high current welded joint. The increase in weld current also increased the weld diameter. The microstructure analysis revealed no phase transformation on the SS316L interface; instead, the significant grain growth occurred. The phase transformation has occurred on the Ti_6_Al_4_V interface. The intermetallic compound layer was also investigated in detail using the EDX (Energy Dispersive X-Ray) and XRD (X-Ray Diffraction) analyses. It was also found that both stainless steel and titanium alloy have their own fusion zone, which is indicated by the highest microhardness value.

## 1. Introduction

Resistance spot welding has been intensively used in automotive and aerospace industries to perform rapid and efficient manufacturing processes. This process aimed to join two pieces of the metal sheet without any additional weight on its joint. Several joining processes, such as riveting and arc welding, place additional weight on the joints, which should be avoided to obtain a good product quality [1,2,3]. Considering the additional weight caused by inefficient joining techniques, a recent study by Costanza et al. investigated explosion welding, that could be an option to join dissimilar materials without additional weight [4]. However, although this method is effective to reduce the additional weight on the joint, the joining process is less effective for the automotive and aerospace industry due to the complex equipment needed and difficulties in defining the optimum welding parameters. In order to gain a good quality and efficient product, the combinations between two dissimilar materials are implemented using the resistance spot welding method. Previous research on dissimilar resistance spot welding has been widely conducted, on materials such as low carbon steel and aluminum alloy, aluminum and titanium, carbon composites and titanium alloy, DP600 steel, and stainless steel [5,6,7,8]. However, stainless steel and titanium alloy are the most commonly used materials in the automotive and aerospace industries. The combination of these two materials has abundant benefits.

The reasons why stainless steel is being commonly used in the industrial sector are its good corrosion resistance, ease of fabrication, high durability, and good weldability. At the same time, titanium alloy also has a lot of benefits, such as the fact of being a super lightweight material, as well as its high corrosion resistance, high strength material, and good weldability [9,10]. However, to obtain a good joint quality between stainless steel and titanium alloy is not easy. Their significant difference in mechanical and thermal properties has become a tremendous challenge for current researchers. The previous study regarding the direct joining process of stainless steel 316L and Ti_6_Al_4_V titanium alloy has been investigated by Mansor et al. The result showed a low joint quality, indicated by a low tensile-shear strength of only 0.395 kN and the appearance of weld defects such as severe micro-cracks on the joint morphology and a brittle fracture on the fracture surface of the joint [11]. Several investigations on the direct SS–Ti joint have been widely conducted by previous researchers. The results showed that the intermetallic compound layer was successfully exhibited on the SS/Ti interface. The diffusion-reaction forced each atom to diffuse and dissolve into the atomic vacant position. Reaction phases such as FeTi, Fe_2_Ti, and Fe_2_Ti_4_O were formed. This phenomenon convinced researchers that the diffusion-reaction and intermetallic compound were successfully obtained on the direct joining process of the SS–Ti joint. However, the direct joining process of SS–Ti is still not recommended by previous researchers [12,13,14,15,16]. Meanwhile, the recommendations to use an interlayer on the SS–Ti joint have been successfully investigated. The application of Nb, Cu, Ni, Ag, and Al have successfully improved the joint quality of the SS–Ti joint [15,17,18]. The other method of interlayer application has been investigated by Winnicki et al. for an aluminum and steel joint by implementing the aluminum, nickel, and nickel-aluminum coating as an interlayer. The coating covered the steel surface by the cold spraying method. The result showed that the joint strength successfully improved compared with the aluminum base material without coating interlayer [19]. Therefore, aluminum has been considered to be the best interlayer for the SS–Ti joint. Besides the lightweight and availability in the market, the titanium alloy grade 5 in this study is also composed of aluminum. Therefore, by implementing the aluminum interlayer, a good joint quality could be achieved.

Although several studies are available in the literature regarding the SS–Ti joint using the resistance spot welding method by implementing a direct joining process or using the interlayer, there is no trace of a detailed study on the effect of welding current and electrode force during the resistance spot welding process. Meanwhile, the weld current and electrode force are important parameters to produce generated heat input on the welded joint. Moreover, previous studies used the small thickness of the base metal, with a thickness of no more than 1 mm. However, the detailed investigation of applied welding parameters on the properties of a 3 mm thick stainless steel and titanium alloy joint, such as heat input, weld diameter, and physical and mechanical properties using the resistance spot welding method can be categorized as a tremendous finding. The joint quality of the resistance spot welded joints of these materials has a great influence in the automotive and aerospace industry. Thus, the resistance spot welding capabilities and possibilities of SS–Ti materials and the possible mechanical and chemical properties of the joint are investigated in this study.

## 2. Materials and Method

In this study, 3 mm thick SS316L and Ti_6_Al_4_V plates were joined using the resistance spot welding method with the application of a 2 mm thick AA5754 aluminum alloy as interlayer. The chemical compositions of SS316L, Ti_6_Al_4_V, and AA5754 alloys are indicated in Table 1.

The SS316L and Ti_6_Al_4_V plates were cut into 114 × 25 mm^2^ according to the ASTM D1002 standard [20] for lap joint configuration. The samples were welded using a DAIDEN AC 220V Spot Welder SL-AJ 35-600 (Osaka, Japan) machine with 9 mm of electrode diameter. For the joining process, the samples were divided into three categories of low current, medium current, and high current. The variations of welding current were 11 kA, 12 kA, and 13 kA followed by the electrode force of 3 kN, 4 kN, and 5 kN, respectively, while the other parameters such as squeeze time, welding time, and holding time were kept constant at 40 cycles, 30 cycles, and 30 cycles, respectively (1 cycle = 0.02 s). According to the welding parameters given, there were three runs of experiments, which are shown in Table 2.

**Figure 1:**
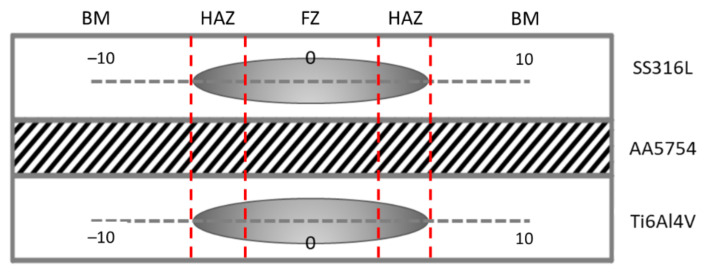
Microhardness measurement configuration.

The tensile-shear load test was carried out using a Zwick/Roell Universal Testing Machine (Bickenbach, Germany) with 50 kN of maximum load capacity and 10 mm/min of ramp speed at room temperature with three times repetition in order to confirm the validity of the results. A metallurgical microscope from LEICA (Wetzlar, Germany) was used to investigate the microstructure morphology of the samples. The microstructure morphologies consist of a base metal (BM), heat affected zone (HAZ), fusion zone (FZ), and an intermetallic compound (IMC) between the SS–Al and Al–Ti interface. Meanwhile, the ZEISS LX0093 Electron Microscope (Oberkochen, Germany) was also used to investigate the chemical composition under the EDX (Energy Dispersive X-Ray) and XRD (X-Ray Diffraction) analyses to take measurements at various points in the welding area. The chemical composition analysis is important to determine the phase formed during the application of Al as an interlayer on the implementation of different welding currents and electrode forces in the welding process. Meanwhile, prior to the microstructure analysis, the cross-sectioned cut was applied to the samples, followed by the grinding and polishing processes to obtain good results. The Carpenters etch was applied to the stainless-steel side, and the Kellers etch was applied to the titanium and aluminum alloy side to achieve good microstructure images. The microhardness measurements were carried out using the LECO LM247AT (St. Joseph, MI, USA) type Vickers microhardness machine under a 200 gf load. The measurements of the samples were taken in one direction along with the BM, HAZ, and FZ of both sides of SS316L and Ti_6_Al_4_V with the 0.5 mm interval of each indentation. The measurement configuration is shown in Figure 1.

## 3. Results and Discussion

### 3.1. Heat Input and Physical Properties Analysis

Resistance spot welding is a thermal joining process involving heat as a major parameter to create a joint between two pieces of metal. According to Joule’s law, the amount of generated heat on resistance spot welding depends on several factors, such as the weld current which flows through the amperage, the welding time which conducts the heating process at a certain time, and the resistance of the materials during the welding process.

**Figure 2:**
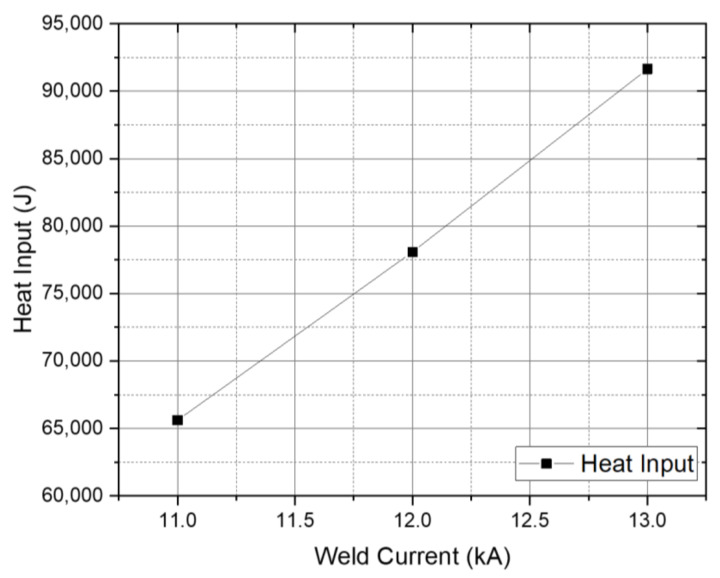
Generated heat input at different welding currents.

The heat input was calculated by the correlation between the test specimens and the electrodes using Equation (1) of the AC (Alternating Current) spot welding heat input calculation, where *Q* is the generated heat input (J), *I* is the welding current (A), *R* is the resistance of the specimens (ρ, electrical resistivity (Ω m) SS316L: 7.4 × 10^−7^, AA5754: 5.22 × 10^−8^, Ti_6_Al_4_V: 17,1 × 10^−7^), and *t* is the welding time (s) [21,22,23,24]. The samples’ resistance in this study was determined using Equation (2). Since this study used dissimilar base metals and interlayer, the sum of three samples’ resistance was taken for the generated heat input calculation. Figure 2 shows the variance of generated heat input observed on each experiment run according to the variance of each welding parameter.

(1) $$Q=0.241\times I^{2}\times \;R\;\times \;t$$

(2) $$R=\frac{\rho \;\times \;L}{A}$$

**Figure 3:**
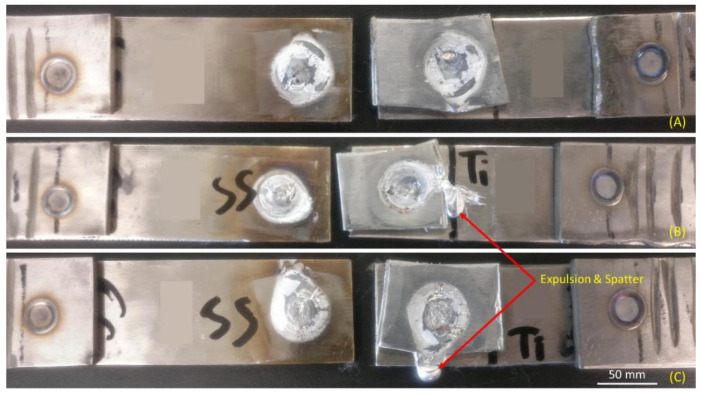
Resistance spot welded joint appearance after tensile-shear test under different generated heat inputs: (**A**) 65.6 kJ; (**B**) 78.1 kJ; (**C**) 91.6 kJ.

As depicted in Figure 2, since the welding current has a quadratic equation on Joule’s law, the increase in the welding current significantly affects the amount of generated heat during the welding process. Although different base metals have different heat increase rates, it does not affect the increase of generated heat on the weld specimens [21,25] [20,24]. As shown in Figure 2, experiment 3 has the highest heat input due to the highest welding current implemented. The 1000 Amperage gap of each experiment run brings a significant increase to the welding heat input, from 65.6 kJ, 78.1 kJ to 91.6 kJ. The heat input significantly affects the weld quality and appearance. Generally, the higher the heat input, the higher the melting materials during the welding process [26]. The over-melted welded joint typically has low joint strength due to the imperfect bonding formation caused by the expulsion that occurred when the high welding current was applied. Figure 3 illustrates the weld appearance of each experiment run.

The weld appearances after the tensile-shear test are shown in Figure 3. From the physical analysis, it could be concluded that the failure for the three experiments was interfacial failure mode, which has the characteristic of brittle failure. The spatter and expulsion have occurred on samples 2 and 3 due to the high welding current implemented. The aluminum interlayer which has the lowest heat resistivity and highest heat input rate showed massive expulsion on sample 2, which is shown in Figure 3B. The expulsion and spatter generally caused the weld joint strength to decrease significantly due to the over-melted interlayer. Then, it caused the bonding between the base metals and the interlayer to be less significant due to the melted aluminum interlayer spreading in every direction and outside the interlayer zone. Thus, the bonding-reaction could not be obtained. Meanwhile, it was also revealed that the highest weld diameter was achieved by sample 3, which has the highest welding current implemented.

**Figure 4:**
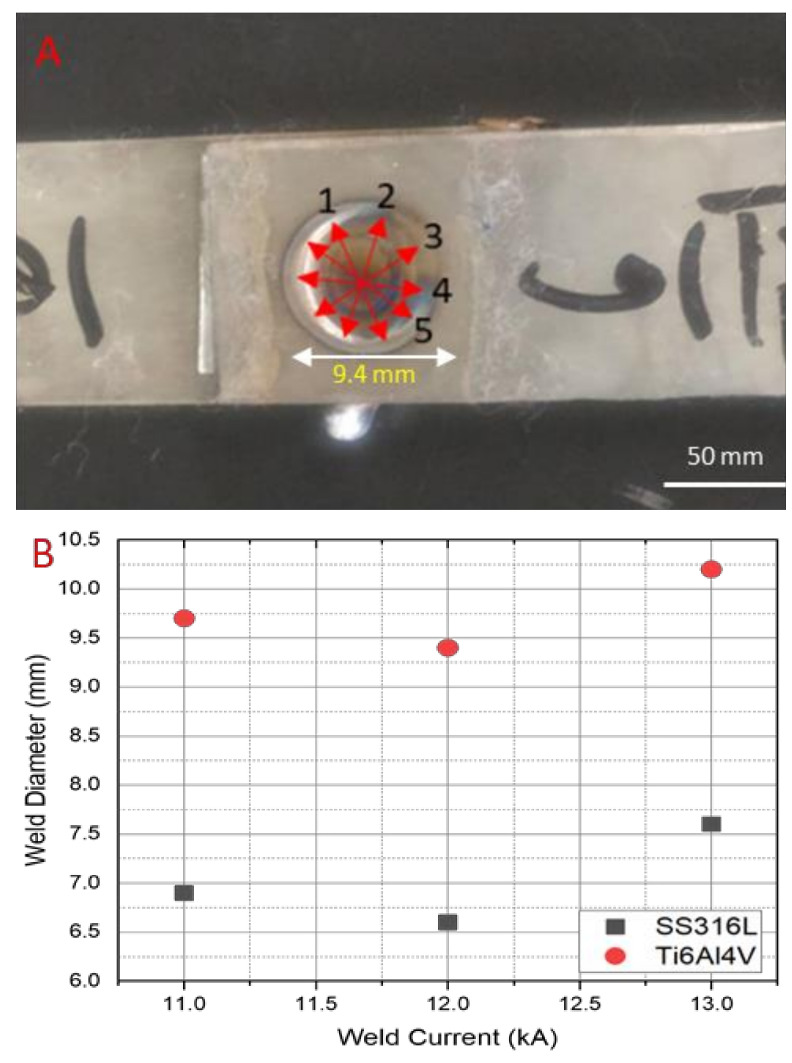
Weld diameter measurements: (**A**) the dispersion of the measurements; (**B**) the variance of the weld diameter measurement results from sample 1, sample 2, and sample 3.

The effect of the high welding current significantly affected the weld diameter of the welded joint owing to the higher heat input generated at a certain time, which produced the excessive heated and melted materials [27]. The weld diameter size of the welded joint of each stainless steel side and titanium alloy side is shown in Figure 4. Since the welded diameter formed was asymmetric, the measurements were taken after five repetitions, and the average of each welded diameter joint was considered as the weld diameter which is shown in Figure 4A,B. A good weld diameter should have a minimum diameter of *d > 4√t*, where *t* is the thickness of the base metal plate. The measured weld diameters were found acceptable above the minimum requirement. These results prove that the welding parameters applied in the welding experiment were chosen appropriately. The fluctuation results in weld diameter measurement were associated with the different amounts of generated heat input. A different weld diameter size of the base metals has occurred, owing to the different thermal resistivity between stainless steel and titanium alloy. The largest weld diameter, of 7.6 mm (SS316L) and 10.2 mm (Ti_6_Al_4_V), was found in sample 3. It has 13 kA of welding current, which delivered 91.6 kJ of heat input during the welding process. Meanwhile, the diameters of sample 1 and sample 2 were 6.9 mm (SS316L); 9.7 mm (Ti_6_Al_4_V), and 6.6 mm (SS316L); 9.4 mm (Ti_6_Al_4_V), respectively. The external factors such as worn electrodes, dirty surfaces of the base metals and interlayer, and electrical instability may have caused the weld diameter to decrease in sample 2.

### 3.2. Tensile-Shear Test

**Figure 5:**
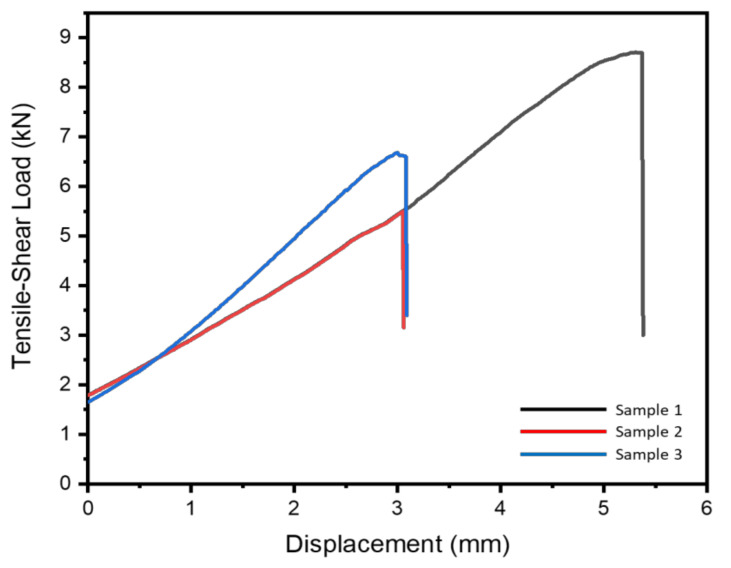
Tensile-shear load graph of the welded samples.

One of the most important findings from the study was that the implementation of various welding currents and electrode forces caused the tensile-shear load results to vary. A tensile-shear load is often used to indicate the welded joint strength. The higher the tensile-shear load, the better the joint strength. The failure mode was also investigated after the tensile-shear test was conducted, which is shown in Figure 3. The failure mode in this study was the interfacial failure mode for all experiment runs. Meanwhile, Figure 5 indicates the correlation between the experiment runs and the tensile-shear load results. Compared with Figure 4, the variation trend between the weld diameter and the tensile-shear load is almost the same.

The highest tensile-shear load was found in sample 1, with a peak load of 8.71 kN. This could be achieved due to the perfect combination of welding current and electrode force. Although the welding current amount was smaller than that of the other experiment runs, the electrode force applied was only 3 kN, which allowed a wider gap to be formed between the base metals. When the gap was formed, the resistance between the base metals increased and allowed a certain amount of heat to be generated during the welding process in order to melt the interlayer and create a strong bonding between the base metals and the interlayer [28]. With the increase of the welding current from 11 kA to 12 kA in sample 2, the tensile-shear load decreased significantly, from 8.71 kN to 5.49 kN. This was due to the welding current having a significant impact on the welded joint quality according to Joule’s law. Consequently, the spatter and expulsion started to occur as shown in Figure 3B. This phenomenon will significantly affect the weld joint strength, whereas the perfect bonding between base metals and the interlayer could not be achieved due to the excessive amount of generated heat input [29]. Meanwhile, sample 3 showed a slight improvement in the welded joint strength, from 5.49 kN to 6.68 kN. The welding current gives a significant impact on the generated heat input during the welding process. However, the 5 kN of electrode force applied played an important role in preventing over-melted materials during the welding process [30]. The application of the 5 kN electrode force successfully suppressed the base metals and interlayer, which decreased the gap between the base metals and interlayer. Thus, the amount of the melted interlayer could be achieved better than that of sample 2. Nonetheless, the spatter still occurred on sample 3, which is shown in Figure 3C, without any massive expulsion.

### 3.3. Welded Joint Morphology

#### 3.3.1. Cross-Sectional Morphology

**Figure 6:**
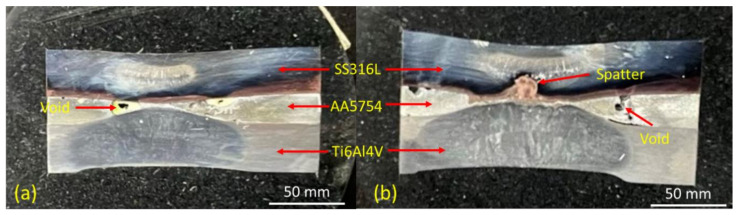
The cross-sectional cut appearance of the welded samples: (**a**) Sample 1-High Tensile-Shear Load; (**b**) Sample 3-High Current.

In this analysis, two samples of sample 1 and sample 3 were taken under the consideration of the highest joint strength (sample 1) and the highest welding current applied (sample 3). Figure 6a,b show the appearance of the welded joint under the cross-sectional cut of sample 1 and sample 3, respectively. The illustrations of the welded joint show that there was a slight difference between sample 1 and sample 3. Both samples have two nugget areas, which consist of a fusion zone from the SS316L side and the Ti_6_Al_4_V side. The effect of the high current and high electrode force applied on sample 3 had a serious impact on the joint morphology. The fusion zone formed was larger than that of sample 1. Moreover, the impact of a high generated heat input and high electrode force successfully squeezes the aluminum interlayer which tends to favor a diffusion-reaction between the base metals and the interlayer. Nevertheless, the effect of high heat input on sample 3 caused the spatter to form in the middle of the interlayer zone. The spatter obviously decreased the joint strength. Moreover, both samples have a void on their cross-sectional cut appearance, which can be categorized as a weld defect. Generally, the internal spatter and void were formed due to the increase of welding current and welding time, which produces a higher generated heat input. With the higher generated heat input, severe weld defects tend to occur rapidly [31,32,33]. However, in this dissimilar resistance spot welding scenario, the weld defect is inevitable. Different thermal properties between the base metals were the main challenge in this research. The optimization of the welding parameters should be further conducted.

#### 3.3.2. Microstructure Examination

**Figure 7:**
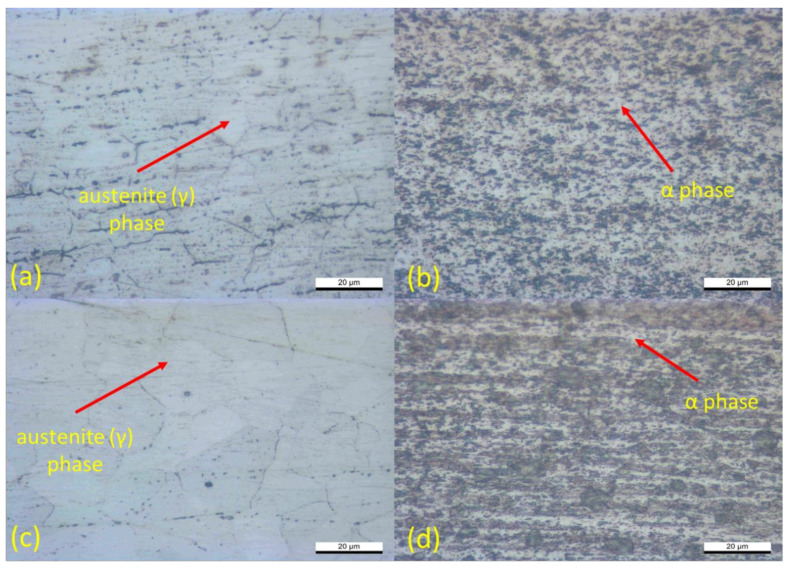
Microstructure of the base metal: (**a**) SS316L-sample 1; (**b**) Ti_6_Al_4_V -sample 1; (**c**) SS316L-sample 3; (**d**) Ti_6_Al_4_V -sample 3.

**Figure 8:**
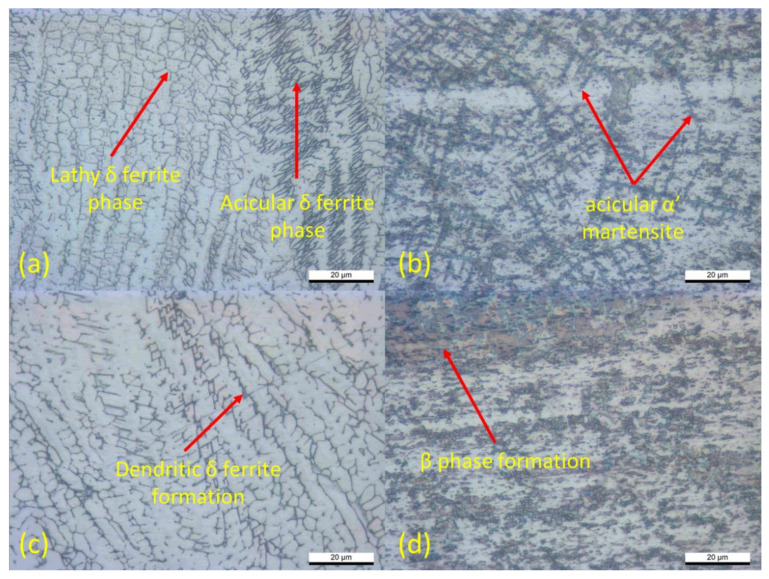
Microstructure of the fusion zone: (**a**) SS316L-sample 1; (**b**) Ti_6_Al_4_V -sample 1; (**c**) SS316L-sample 3; (**d**) Ti_6_Al_4_V -sample 3.

In order to analyze the effect of welding current on the welding morphology, the microstructure analysis is mandatory. The inhomogeneous microstructure of the welded joint was observed due to the variation of heat input and different cooling rates of the investigated samples during the welding process. Figure 7 and Figure 8 show the microstructure morphology from sample 1 and sample 3 at the base metals and fusion zones area. Figure 7a shows the base metals’ microstructure of the SS316L from sample 1, and Figure 7c shows the SS316L from sample 3. Both samples show a similar morphology, which consists of the dominant austenite (γ) phase and less amount of the delta-ferrite (δ) phase on the base metal. The presence of the dominant austenite (γ) phase indicates a lack of generated heat on the base metals for both sample 1 and sample 3. Meanwhile, Figure 7b,d show the Ti_6_Al_4_V base metals morphology from sample 1 and sample 3, respectively. Generally, the titanium alloy used in this study has a dual-phase, which consists of an α phase and a β phase. The presence of 6 wt % aluminum acts as an α phase stabilizer by increasing the α–β transformation temperature. Moreover, the presence of 4 wt % vanadium acts as a β phase stabilizer. These factors render the Ti_6_Al_4_V more feasible for industrial applications. As shown in Figure 7b,d, the α phase dominates the base metal surfaces, which means that less heat was generated on the base metal. However, the presence of the β phase was distributed evenly and was slightly more dominant in sample 3, which is shown in Figure 7d. This is because sample 3 received higher generated heat input during the welding process, which may heat the base metal zone in sample 3.

Figure 8a,c depict the microstructure morphology of the SS316L fusion zone from sample 1 and sample 3, respectively. The transformation of the delta-ferrite (δ) grain to δ vermicular grain has occurred in the transition zone from the base metal to the fusion zone. Moving onto the fusion zone, the δ vermicular grain became more assertive, covering the austenite phase. Since the highest generated heat input was located in the fusion zone, the δ vermicular grain transformed into the δ reticular morphology. Thus, the dominant phase on the fusion zone was the ferritic (δ) and sigma (σ) phase due to the highest generated heat input. Consequently, there is no significant phase transformation on the microstructural analysis of the SS316L side; instead, the grain growth of narrow and coarse delta-ferrite (δ) phase was exhibited. Figure 8a shows the formation of the coarse ferritic grain, which has a more dominant amount and narrower formation compared with Figure 8c. Sample 1 shows the dominant lathy delta-ferrite (δ) grain with the combination of the acicular delta ferrite grain. The formation of acicular delta-ferrite was inevitable since the cooling rate increased significantly due to the short welding time. This means that sample 1 has better-generated heat distribution on the fusion zone which affects the joint quality. Meanwhile, Figure 8c shows the dendritic formation of the coarse delta-ferrite (δ) phase. Convincingly, both sample 1 and sample 3 exhibited a significant grain growth from the base metal to the fusion zone due to the applied welding current and electrode force. Previous investigations showed similar morphology on the stainless steel microstructure analysis during the resistance spot welding process [34,35]. On the other hand, the microstructure of Ti_6_Al_4_V for sample 1 and sample 3 has been investigated and shown in Figure 8b,d. Both samples exhibited the coarsened grain structure due to the room-temperature cooling rate. During the welding process, the various morphologies of primary α, primary β, and the transformation of the β phase to some fine acicular α’ phase were found on the Ti_6_Al_4_V alloy side. The microstructure appearance of sample 1, which is shown in Figure 8b, showed that the presence of β phase was entirely transformed into coarse acicular α’ martensite phase covering the primary α phase. Meanwhile, less formation of acicular α’ martensite was exhibited in sample 3, and the narrower β phase formation has occurred instead. This may occur due to the excessive generated heat input having been implemented on sample 3 during the welding process. The difference in microstructure morphology will significantly affect the joint quality between the two samples.

#### 3.3.3. The Effect of Welding Parameters on the Interlayer

**Figure 9:**
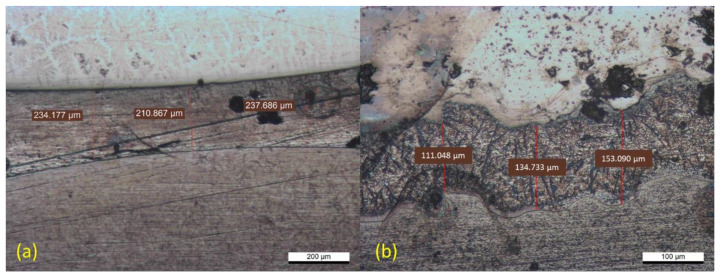
Aluminum interlayer morphology: (**a**) Sample 1—weld current: 11 kA, electrode force: 3 kN; (**b**) Sample 3—weld current: 13 kA, electrode force: 5 kN.

Further investigation has been conducted to evaluate the effect of welding parameters on the applied interlayer. In this study, the various amounts of welding current and electrode force had a significant impact on the applied interlayer behavior during the welding process. Figure 9 shows the morphology of the aluminum interlayer under different welding parameters, with an image accuracy up to 1 µm. A perfect drum-shaped nugget was observed in sample 1, which is shown in Figure 9a. The thinnest interlayer was observed at the middle of three measurements, with a thickness of 210.867. Surprisingly, the combination of the 11 kA welding current and the 3 kN electrode force tended to form a perfect drum-shaped interlayer. This means that the generated heat input on sample 1 was perfectly distributed along the welded area. A low amount of electrode force also allows heat input to be distributed perfectly and prevent any excessive heat on the specific welded area. Therefore, the intermetallic compound layer and diffusion-reaction between stainless steel-aluminum and aluminum-titanium alloy could be achieved effectively. Meanwhile, Figure 9b shows the morphology of the aluminum interlayer from sample 3, with 13 kA of welding current and 5 kN of electrode force. The high amount of welding current and electrode force brought a massive impact to the interlayer morphology of sample 3. As shown in Figure 9b, the interlayer appearance was extremely different than that in sample 1. The interlayer thickness was much thinner than in sample 1. The thinnest interlayer, of 111.048 µm, was measured along with the aluminum interlayer nugget. The interlayer on sample 3 was extremely suppressed owing to the implementation of high welding current and electrode force. The high amount of electrode force causes excessive heat to be distributed massively at the specific region of the welded area. Thus, the aluminum interlayer which has the lowest melting point compared with the other base metals was conveniently melted and suppressed into an asymmetric aluminum interlayer formation. However, the joint strength was significantly decreased due to the excessive amount of melted aluminum interlayer, and therefore the diffusion-reaction between the base metals and interlayer could not be achieved perfectly.

### 3.4. Chemical Composition Analysis

During the resistance spot welding of SS316L and Ti_6_Al_4_V using an aluminum interlayer, the diffusion-reaction has occurred owing to the intermetallic compound layer formation. Stainless steel, titanium alloy, and aluminum alloy have a close atomic radius number of Al-184, Ti-187, and SS-194 pm, respectively. During the heating process from the electrode, the atoms from each material were moving in any direction. Therefore, the atomic vacancy diffusion was inevitable due to each material having a close atomic radius number. Vacancy diffusion is one mechanism that involves the interchange of an atom from a normal lattice position to an adjacent vacant lattice site [36].

**Figure 10:**
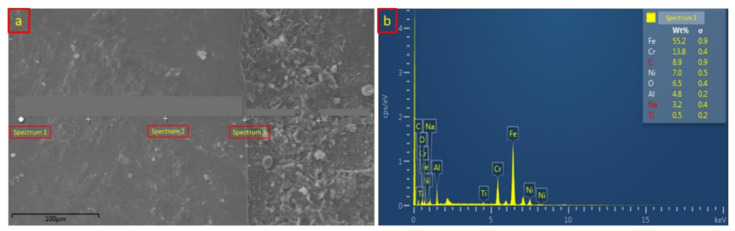
(**a**) SS/Al spectrum distribution of the EDX analysis from sample 1; (**b**) SS/Al EDX analysis result on the IMC layer from sample 1.

The diffusion-reaction between the base metals and the interlayer is necessary for this study to obtain a good welding quality. Table 3 and Figure 10 show the chemical composition of sample 1 from the EDX analysis of the SS/Al interface. Spectrums 1, 2, and 3 represent the position of EDX analysis distribution as shown in Figure 10a. Spectrum 1 shows the chemical composition of the stainless steel 316L at the base metal zone. It shows that the dominant composition of 70.1 wt % Fe still exists and that both Al and Ti compositions were not found in this zone, which means there was no generated heat input on the base metal zone from sample 1. Moving onto spectrum 2, which is closer to the intermetallic compound (IMC) layer, the composition of Fe decreased, and new chemical compositions of C, Na, and Cu were found. This area showed that the lower generated heat input had a slight effect on the chemical composition of the base metal. The interaction between stainless steel and aluminum alloy happened in the intermetallic compound which is represented in spectrum 3. The chemical composition recorded a lower amount of 4.8 wt % aluminum and 0.5 wt % titanium, which means that the generated heat input successfully conceived a bonding reaction between the stainless steel and aluminum alloy. Figure 10b shows the EDX analysis result at the intermetallic compound of the SS/Al interface.

**Figure 11:**
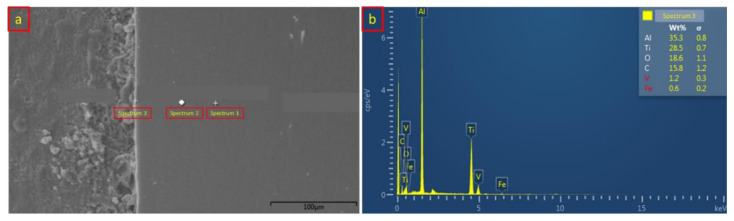
(**a**) Ti/Al spectrum distribution of the EDX analysis from sample 1; (**b**) Ti/Al EDX analysis result on the IMC layer from sample 1.

On the other hand, Table 4 and Figure 11 represent the chemical composition analysis of the titanium and aluminum alloy interface of sample 1. The EDX analysis distribution was similar to that of the SS/Al interface, which is shown in Figure 11a. At the base metal zone, spectrum 1 indicated the highest composition of 91.0 wt % titanium followed by the aluminum and vanadium, which confirmed the implementation of Ti_6_Al_4_V titanium alloy. Spectrum 2 shows that a slight composition of carbon (C) has appeared due to the generated heat input. Meanwhile, spectrum 3 shows the chemical composition of the Ti/Al IMC layer. According to the EDX analysis, the composition of aluminum increased significantly, from 5.6 wt % to 35.3 wt %, while the composition of titanium remains at 28.5 wt %. The diffusion-reaction between titanium and aluminum alloy has a better quality and stronger bonding due to the close atomic radius number, which causes a vacancy diffusion to efficiently occur. Figure 11b shows the EDX analysis result from spectrum 3, which depicts the IMC layer of the Ti/Al interface.

**Figure 12:**
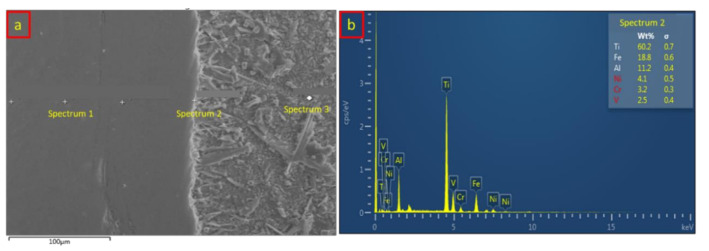
(**a**) SS/Al spectrum distribution of the EDX analysis from sample 3; (**b**) SS/Al EDX analysis result on the IMC layer from sample 3.

A chemical composition analysis has also been done for sample 3, implementing the application of a high current and high electrode force. The effects of both parameters were further investigated in this study compared with the chemical composition and diffusion-reaction behavior from sample 1. Table 5 and Figure 12 show the chemical composition and EDX analysis of the SS/Al interface, which consists of the base metal, the IMC layer, and the interlayer area of analysis. Figure 12a shows the spectrum distribution, and the chemical compositions are listed in Table 5. Spectrum 1 shows the dominant composition of Fe which indicated the base metal of SS316L. An uncommon phenomenon has been observed where the composition of Ti and Al slightly appeared at the SS316L base metal with the composition of 14.2 wt % and 1.4 wt %, respectively. Meanwhile, the presence of Ti and Al composition was not found in sample 1. This is due to the inadequate welding current and electrode force during the resistance spot welding process of sample 1 [37]. The application of 13 kA and 5 kN of welding current and electrode force in sample 3 forced the titanium and aluminum to be diffused through the base metal of SS316L. Spectrum 2 shows the EDX location at the IMC layer of the SS/Al interface. Surprisingly, the composition of titanium significantly increased from 14.2 wt % to 60.2 wt % at the IMC layer of the SS/Al interface. It is obvious that the titanium has a better diffusivity and solubility to aluminum and stainless steel under the high welding current and high electrode force. Considering that the interlayer thickness was relatively small in this sample, the composition of Ti and Al could be easily diffused through the stainless steel side. The composition of Al also increased from 1.4 wt % to 11.2 wt %, which proves that the IMC layer at the SS/Al interface has been successfully formed. Meanwhile, spectrum 3 indicated the chemical composition of the aluminum interlayer, which consists of a dominant composition of Al and a small amount of 4.3 wt % Fe. Figure 12b depicts the EDX analysis result from the IMC layer.

**Figure 13:**
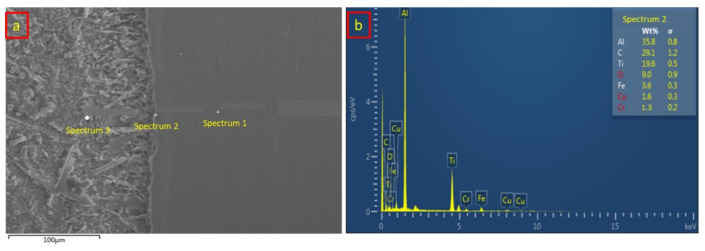
(**a**) Ti/Al spectrum distribution of EDX analysis from sample 3; (**b**) Ti/Al EDX analysis result on the IMC layer from sample 3.

Table 6 and Figure 13 illustrate the chemical composition and EDX analysis from the Ti/Al interface. The spectrum distribution and analysis scheme were similar to those of the SS/Al interface which is shown in Figure 13a. According to Table 6, spectrum 1 shows the dominant composition of the Ti_6_Al_4_V base metal with an additional slight amount of 3 wt % carbon. The composition of Ti was significantly decreased on spectrum 2 followed by the increase of Al from 5.5 wt % to 35.8 wt %, which shows that the diffusion-reaction between titanium and aluminum alloy was successfully obtained at the IMC layer. Additionally, the composition of 3.6 wt % Fe appeared at the IMC layer of the Ti/Al interface. The applied high welding current and high electrode force successfully pushed the Fe to be diffused further to the IMC layer of the Ti/Al interface. Spectrum 3 indicated the dominant composition of aluminum, with 76.6 wt % of atomic weight.

Comparatively, sample 3, which implemented the high welding current and high electrode force, brought a significant amount of diffusion-reaction between the base metals and interlayer compared with sample 1. The composition of Ti successfully reached the SS/Al IMC layer, and the Fe composition reached the Ti/Al IMC layer. However, due to the application of high welding current and high electrode force, massive expulsion has occurred, and the interlayer thickness decreased and was significantly suppressed. Therefore, the joint strength and quality of sample 3 have decreased simultaneously.

**Figure 14:**
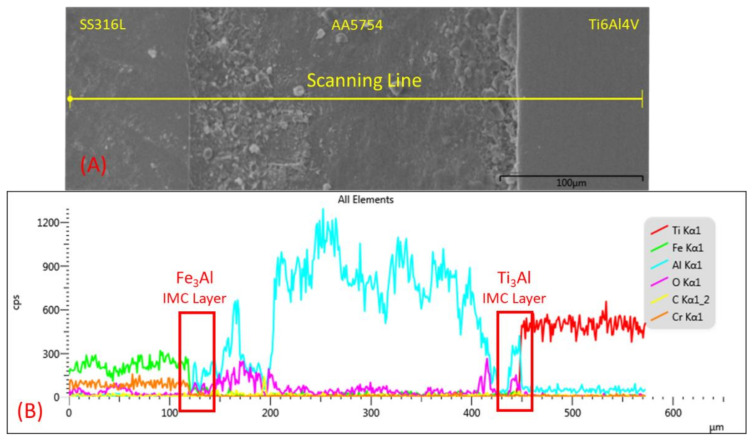
Fe, Al, and Ti distributions across the SS–Al–Ti joint interface from sample 1: (**A**) EDX scanning line; (**B**) chemical composition along the scanning line distribution.

According to the EDX scanning line from sample 1 and sample 3, the diffusion-reactions clearly occurred along the welded joint interface, especially at the intermetallic compound layer between the SS/Al and Ti/Al interfaces. Figure 14 shows the chemical composition from the EDX scanning line of sample 1. The result showed that the Al composition was successfully diffused and blended into the Fe composition. Thus, the Fe_3_Al compound was formed under the diffusion-reaction of the SS/Al interface according to the Fe–Al binary phase. On the other hand, the Ti_3_Al compound was also formed on the Ti/Al IMC layer according to the Ti–Al binary phase. According to the EDX scanning result, the aluminum composition successfully bonded with the titanium, which is indicated by the blue and red line in Figure 14. The bonding between titanium and aluminum caused the joint quality of the SS–Ti resistance spot welded to increase significantly [38].

**Figure 15:**
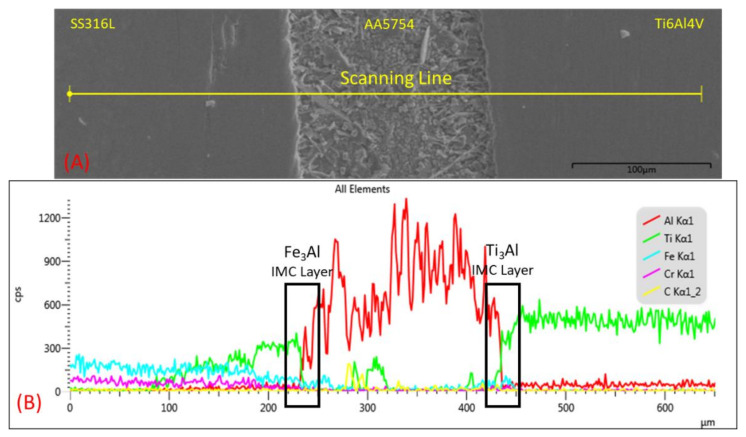
Fe, Al, and Ti distributions across the SS–Al–Ti joint interface from sample 3: (**A**) EDX scanning line; (**B**) chemical composition along the scanning line distribution.

Surprisingly, sample 3 has a similar compound formation of Fe_3_Al and Ti_3_Al, which is shown in Figure 15. A high current and high welding force implemented on sample 3 led to a significant amount of diffused titanium composition. The composition of titanium successfully broke through the SS/Al IMC layer which is indicated by the green line in Figure 15. This phenomenon happened due to the high heat input causing the interlayer to be over-melted and the high electrode force successfully suppressing the interlayer to form a thinner interlayer thickness compared with sample 1′s interlayer. Therefore, since titanium has a higher electrical resistivity, which means that it has a better heat conductivity, the atom of titanium could easily diffuse to the stainless steel atom. The direct diffusion of Fe and Ti could decrease the joint quality of the SS–Ti joint. A previous study investigated a direct joining process using the resistance spot welding of SS316L and Ti_6_Al_4_V without any interlayer. The results showed that the joint strength was not higher than 0.4 kN. Moreover, the joint morphology was a fully brittle failure, and severe micro-cracks were observed under the microstructure analysis [10]. Therefore, a direct joining process of the SS–Ti joint should be avoided.

**Figure 16:**
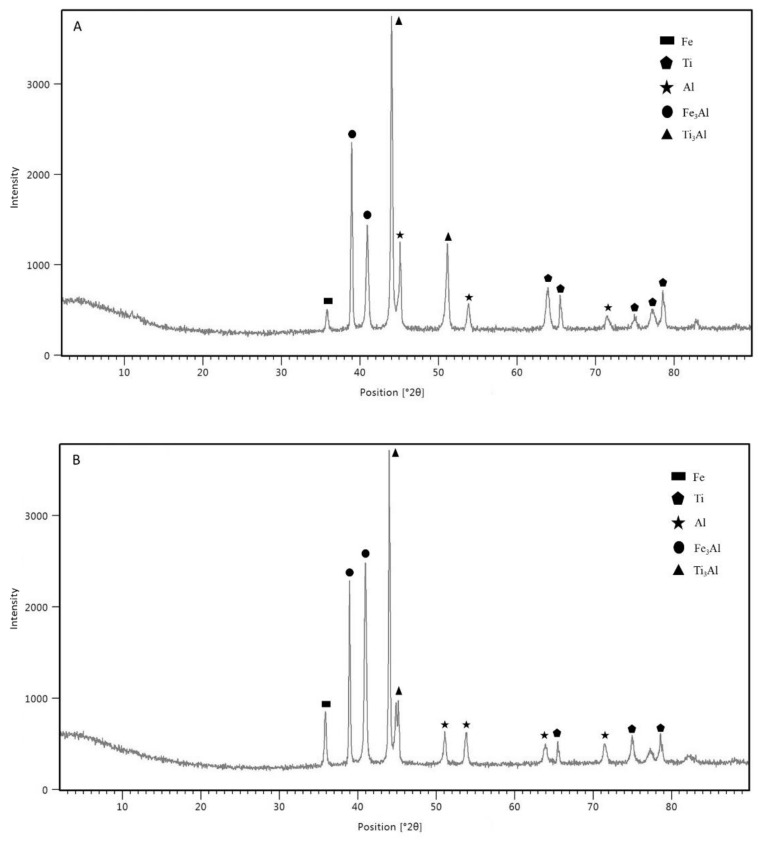
XRD result along the SS–Al–Ti joint cross-sectional cut: (**A**) sample 1; (**B**) sample 3.

The intermetallic compound layer formation has an important role in this study. The possible phase formation defines the diffusion-reaction between the base metals and the interlayer. SS/Al and Ti/Al have their own IMC layer, which was successfully formed under the resistance spot welding process. According to the Fe–Al binary phase diagram, the possible phases formed are FeAl, Fe_3_Al, FeAl_2_, Fe_2_Al_5_, and FeAl_3_. Meanwhile, based on the Ti–Al binary phase diagram, the possible phases formed are TiAl, Ti_3_Al, TiAl_2_, and TiAl_3_ [15]. The XRD analysis was conducted in order to confirm the phase formed on the IMC of SS/Al and Ti/Al. The results are shown in Figure 16A for sample 1 and Figure 16B for sample 3 XRD analysis. There was no significant difference between sample 1 and sample 3 where the presence of Fe_3_Al and Ti_3_Al was confirmed and appeared dominant. However, due to the application of high welding current and high electrode force in sample 3, the compositions of the Fe_3_Al phase and aluminum were higher than in sample 1.

### 3.5. Microhardness Test

**Figure 17:**
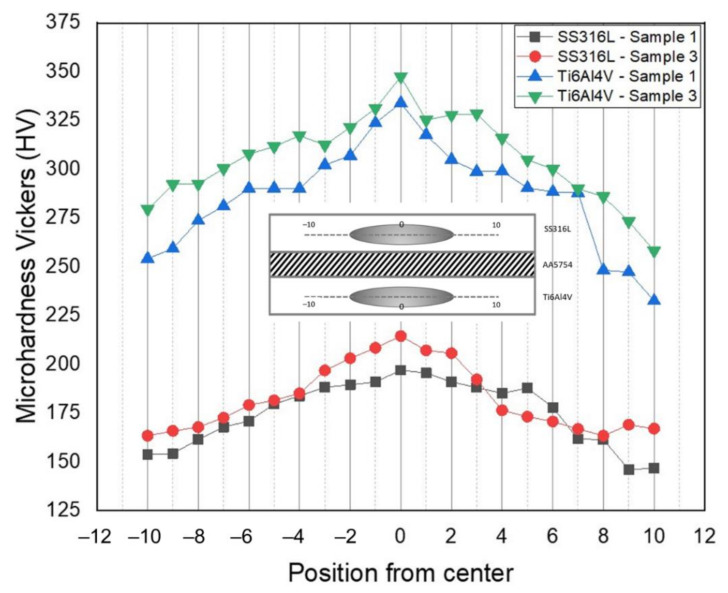
Microhardness Vickers profile from the SS–Ti joint with an aluminum interlayer.

Figure 17 illustrates the microhardness distribution of the SS–Ti resistance spot welded joint, using Microhardness Vickers (HV), gained with different welding current and electrode force. Since both the SS and Ti interfaces exhibited their own weld nugget, the microhardness distributions were taken horizontally along each of the stainless steel and titanium sides for both sample 1 and sample 3. Generally, the microhardness distribution was divided into three areas—base metal (BM), heat affected zone (HAZ), and fusion zone (FZ)—with an accuracy of up to 1 HV unit. The lowest hardness value was located at the base metal and the highest one at the fusion zone. The results showed that the microhardness of sample 3 had a higher value than that of sample 1 due to the higher heat input received during the resistance spot welding process. The microhardness value of the base metal was 153.8 HV for sample 1 and 163.4 HV for sample 3. Moving onto the heat-affected zone where the heat was affecting the base metal and causing grain growth to occur, the microhardness value increased from 161.5 HV to 171 HV for sample 1 and 172.7 HV to 179.1 HV for sample 3. The highest generated heat input was located at the fusion zone, which caused the microstructure morphology to change into lathy and acicular delta-ferrite (δ) phase. However, the microhardness value increased significantly, becoming 197 HV for sample 1 and 214.5 HV for sample 3.

Meanwhile, the microhardness value of the titanium side’s base metal was measured at 254.2 HV for sample 1 and 279.5 HV for sample 3. The heat-affected zone which had the dominant β phase due to the less generated heat input produced the microhardness value of 281.2 HV for sample 1 and 300.4 HV for sample 3. The highest microhardness value was also located at the fusion zone of the titanium side which had the microstructure formation of α’ martensite phase due to the massive heat input and rapid cooling rate. The phase transformation of titanium causes the microhardness value to increase significantly. The highest microhardness value was recorded at 334 HV for sample 1 and 347.5 HV for sample 3. Conclusively, the comparison between low heat input and high heat input which was implemented on sample 1 and sample 3 delivered a significant difference on the microhardness value. Higher heat input causes a higher microhardness value due to the significant phase transformation of each material.

## 4. Conclusions

The current study showed that a good quality of SS–Ti resistance spot welded joint with aluminum interlayer could be achieved by considering the effect of welding parameters such as welding current and electrode force which have been investigated in this study. Therefore, the following conclusions can be drawn:

1. As the welding current and the electrode force increased, the generated heat input on the resistance spot welded samples also increased. The effect of a higher generated heat input affected several joint qualities, such as a greater weld nugget diameter, massive spatter and expulsion on the highest heat input, microhardness value, and the microstructure and chemical composition of the entire joint morphology. Meanwhile, the highest joint strength of 8.71 kN was achieved at the lowest heat input of the 11 kA welding current, 3 kN electrode force, and 30 cycles of welding time and holding time.
2. High welding current and electrode force also affected the microstructure morphology. The lathy and acicular delta-ferrite (δ) phases were found in the low heat input sample at the stainless steel interface. Meanwhile, the dendritic delta-ferrite (δ) phase was found to be more dominant at the high heat input of the stainless steel interface. The titanium morphology consisted of acicular α’ martensite and a dominant β phase transformation, which is the primary cause of the increase of the microhardness value. Meanwhile, the interlayer thickness significantly decreased due to the applied high welding current and high electrode force. The higher heat input caused the over-melted aluminum interlayer, and the higher electrode force convincingly suppressed the interlayer to the smaller thickness. These factors lead to a decrease in joint strength and joint quality.
3. The chemical composition analysis showed that the higher welding current and electrode force are prone to produce better diffusion-reaction between the base metals and the interlayer. The formation of Fe_3_Al and Ti_3_Al were successfully exhibited at the intermetallic compound layer of the SS/Al and Ti/Al interfaces for both samples. Additionally, the diffusion-reaction rate at sample 3, which was welded with the highest welding current and highest electrode force, was found to be significantly higher than that of the samples welded with low welding current and electrode force.
4. The microhardness value was found to be relatively higher at the sample which implemented the highest welding current and electrode force. The excessive generated heat input and rapid cooling temperature, that caused microstructural transformation, led to the increase of the microhardness value. The highest microhardness value in this study was recorded at 214.5 HV of the stainless steel side and 347.5 HV of the titanium alloy side.

## Figures and Tables

**Table 1:** Chemical composition of SS316L, Ti6Al4V, and AA5754 alloy (wt %).

Element	Cr	Ni	Mn	Mo	Fe	Al	Ti	V	C	O	N	Mg
**SS316L**	17.68	12.6	1.53	2.38	Bal.	0.018	0.021	0.663	0.016	-	-	-
Ti_6_Al_4_V	-	-	-	-	0.4	6.75	Bal.	4.5	0.08	0.02	0.05	-
**AA5754**	0.3	-	0.5	-	0.4	93.6	0.15	-	-	-	-	3.6

**Table 2:** Experimental order.

Welding Parameter	Weld Current (kA)	Welding Time (Cycles)	Holding Time (Cycles)	Electrode Force (kN)
Sample 1	11	30	30	3
Sample 2	12			4
Sample 3	13			5

**Table 3:** EDX analysis results of the SS/Al intermetallic compound (IMC) layer from sample 1.

Spectrum	Composition (wt %)								
	Fe	Cr	Ni	O	C	Al	Na	Cu	Ti
1	70.1	17.8	10.7	1.4	-	-	-	-	-
2	40.6	12.2	5.8	13.6	9.7	-	9.3	8.8	-
3	55.2	13.8	7.0	6.5	8.9	4.8	3.2	-	0.5

**Table 4:** EDX analysis results of the Ti/Al IMC layer from sample 1.

Spectrum	Composition (wt %)					
	Ti	Al	V	C	O	Fe
1	91.0	5.4	3.6	-	-	-
2	87.5	5.6	3.6	3.3	-	-
3	28.5	35.3	1.2	15.8	18.6	0.6

**Table 5:** EDX analysis results of the SS/Al IMC layer from sample 3.

Spectrum	Composition (wt %)						
	Fe	Ti	Cr	Ni	C	Al	V
1	55.2	14.2	14.2	8.8	6.2	1.4	-
2	18.8	60.2	3.2	4.1	-	11.2	2.5
3	4.3	-	1.5	-	13.8	80.4	-

**Table 6:** EDX analysis results of the Ti/Al IMC layer from sample 3.

Spectrum	Composition (wt %)							
	Ti	Al	V	C	O	Fe	Cu	Cr
1	87.4	5.5	4.1	3.0	-	-	-	-
2	19.6	35.8	-	29.1	9.0	3.6	1.6	1.3
3	0.6	76.6	-	18.3	2.7	1.8	-	-

## Data Availability

Data is contained within the article.
